# Water Footprint Allocation under Equity and Efficiency Considerations: A Case Study of the Yangtze River Economic Belt in China

**DOI:** 10.3390/ijerph16050743

**Published:** 2019-03-01

**Authors:** Gang Liu, Weiqian Wang, Kevin W. Li

**Affiliations:** 1 State Key Laboratory of Hydrology of Water Resources and Hydraulic Engineering, Hohai University, Nanjing 210098, China; lglhm@msn.com; 2 Institute of Management Science, Hohai University, Nanjing 210098, China; wwq@hhu.edu.cn; 3 Hohai University Coastal Development and Protection Collaborative Innovation Center, Nanjing 210098, China; 4 College of Economics and Management, Fuzhou University, Fuzhou 350116, China; 5 Odette School of Business, University of Windsor, Windsor, ON N9B 3P4, Canada

**Keywords:** Yangtze River Economic Belt, water footprints, lexicographic algorithm, equity and efficiency, input-output capacity of water footprints

## Abstract

From a water footprint perspective, this paper adopts Gross Domestic Product (GDP) as the influencing factor to construct a lexicographical optimization framework for optimizing water resources allocation under equity and efficiency considerations. This approach consists of a lexicographic allocation of water footprints (LAWF) model and an input-output capacity of water footprints (IOWF) model. The proposed methodology is then applied to allocate water resources in the Yangtze River Economic Belt (YREB) by employing the 2013 cross-sectional data in the area. The results show that: (1) The LAWF scheme signifies reductions in water footprints in each of the YREB administrative units, thereby significantly strengthening their IOWFs. (2) IOWFs are affected by industrial attributes and natural endowments, and the impact tends to vary across different industries and regions. (3) Policy suggestions are proposed to effectively enhance the IOWFs of the weakest industries across the three YREB regions to exploit their natural endowments.

## 1. Introduction

Using water resources in a sustainable way and improving water use efficiency are two integral aspects of alleviating environmental and ecological stresses and enhancing human welfare. China’s water consumption per 10,000 United States dollar (USD) of Gross Domestic Product (GDP) stands at 1.7 times the world average [1], indicating a significantly low water use efficiency in China. For the Yangtze River Economic Belt (YREB), its population and economy both exceed 40% of the national totals, and its continued population and economic growth has further increased consumption of water resources and exacerbated water shortage in this region. In the meantime, the “Guidelines on Promoting the Development of the YREB Building on the Golden Waterway” points out that the development of the YREB is a national core strategy, so it is essential to guarantee the supply of water resources in this area. Furthermore, water consumption per 10,000 USD of GDP in the YREB is as high as 1.78 times the world average [2], signifying that water use efficiency in the YREB is far below the world average. Considering the dire situation of water resources supply and demand in the YREB, the State Council issued an “Outline for the Development of the YREB” in September 2016, clearly stating that the total water consumption by 2020 should be controlled to within 300 billion m^3^ and the water consumption per 10,000 USD of GDP should be reduced by more than 27% from the 2015 level. All in all, unbalanced water supply and demand and low water use efficiency have become important bottlenecks restricting sustainable development of the YREB. Therefore, properly managing water demand and improving water use efficiency are two key measures to promote sustainable development of the YREB.

The water footprint theory provides us with a novel way to manage water demand. Hoekstra and Hung [3] introduced the concept of water footprints, aiming to account for the total amount of water resources consumed by all products and services in an area within a certain period of time. In general, the notion of water footprints furnishes an innovative perspective on judging water resources consumption that comes close to the actual needs of human beings. Current studies of water footprints mainly consist of three types: the first is to calculate water footprints in production and consumption activities. For example, Mekonnen and Hoekstra [4] determined water footprints of the world’s major livestock products from 1996 to 2005. Chapagain and Hoekstra [5] computed water footprints of rice cultivation. Sun et al. [6] estimated water footprints of different regions in China from 1997 to 2007. The second type of research is to evaluate water footprints from the perspectives of environmental, social, and economic sustainability. For instance, Chapagain and Orr [7] assessed the social and economic impact of water footprints in food and fibre consumptions in the UK while Oel et al. [8] measured the impact of Dutch’s external water footprints on other countries. Casolani et al. [9] investigated water and carbon footprints in Italian durum wheat cultivation. Cartone et al. [10] analyzed the spatial effect on the grey water indicator in crop production. Miglietta et al. [11] used grey water footprints as an indicator to assess the impact of pollution caused by human activities. The third type of research is to examine the transfer process of water footprints. Along this line, Yang et al. [12] considered the efficiency of virtual water trade behind the international food trade. Seekell et al. [13] revealed that virtual water transfer is unlikely to address inequality in global water use. Zhuo et al. [14] discussed the effect of interannual variability of consumption, production and climate on inter-regional virtual water trade. The aforesaid research on water footprints provides the basis for optimizing water resources allocation from a lifecycle perspective. On the other hand, water is a quasi-public resource and its allocation should consider fairness from different aspects. As such, this research aims to consider equitable water resources allocation from a water footprint angle.

Water footprint allocation is a typical multi-agent single-resource allocation problem. To solve this problem with fairness concerns, we can refer to the method of an ordered weighted averaging (OWA) aggregation operator introduced by Yager [15]. In multi-objective decision making with finite stakeholders, the OWA aggregation handles ranking order generated by a lexicographic ranking approach and finds a solution by repeatedly solving the problem with a minimax objective function, which presumably reaches a Pareto-optimal solution with fairness concerns. Scholars have studied fair allocation of resources by using this lexicographic algorithm. For instance, a successive lexicographic minimax algorithm was put forward by Luss and Smith [16] to handle the equitable raw material distribution problem in large-scale industrial production. Based on the lexicographic theory, Wang et al. [17] improved a lexicographic algorithm to examine the allocation of water rights in the Amu River Basin. Betts et al. [18] employed a lexicographic algorithm to solve the complex order constraint problem in the high-tech industry. Luss [19] applied a lexicographic algorithm to fairly allocate bandwidth in a content distribution network. Liu et al. [20] used the Taihu Basin in China as an example to construct a lexicographic quota allocation model for equitably allocating initial emission rights. Recently, Liu et al. [21] developed a lexicographic optimization model to allocate agricultural and non-agricultural water footprints by using the land area as the influencing factor. In addition, some scholars have studied fair allocation of multi-agent single-resource based on different economic indicators. For instance, Pu et al. [22] investigated how GDP affects sustainable water resources governance in a lake basin. Sušnik [23] took a holistic approach to analyze the relationship between GDP growth and the water-energy-food resources. Yin [24] assessed the environmental efficiency and its determinants in western China by accounting for economic growth and other undesirable outputs. Their research reveals that it is an effective way to accommodate fairness and efficiency by using economic indicators to allocate resources.

In summary, analysing water demand from a water footprint perspective provides a new direction for optimizing water resources allocation and improving water use efficiency. However, it remains a challenge to achieve an equitable and effective allocation of water resources. Since existing research proves the lexicographic algorithm to be a useful tool for fair allocation of limited resources, we adopt it as the basis and employ GDP as the influencing factor to construct a lexicographic allocation of water footprints (LAWF) model to reduce water demand in the YREB. Subsequently, we propose an input-output capacity of water footprints (IOWF) model to assess the effectiveness and reasonableness of the allocation scheme. Finally, we further analyse the factors affecting the IOWFs of the LAWF scheme in the YREB and explore how different regions across the YREB can better improve their IOWFs by exploiting their industrial attributes and location characteristics.

## 2. Case Study of the YREB Water Resources Allocation

### 2.1. Background Information

**Figure 1:**
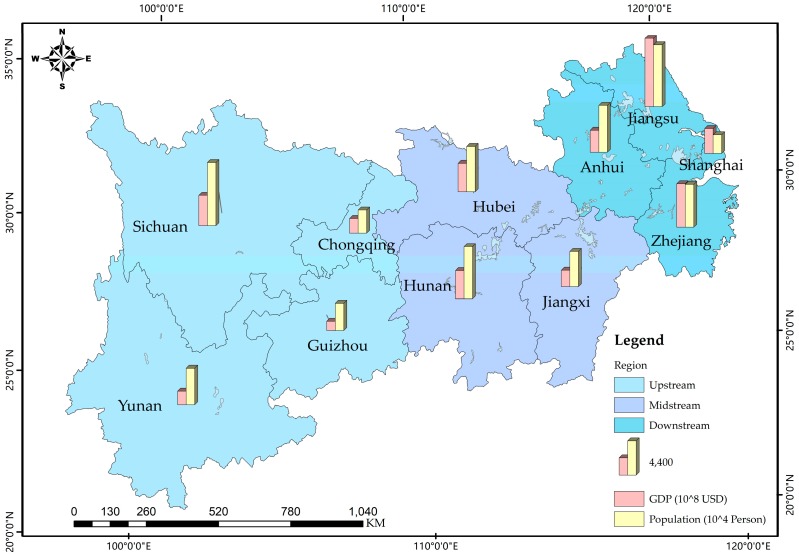
The YREB area in China.

The YREB links China’s western, central and eastern regions and consists of nine provinces and two provincial-level municipalities in the Yangtze River Basin except for Qinghai and Tibet as illustrated in Figure 1. As of 2013, the total population of the YREB reached 588 million and the total annual GDP reached 3.85 trillion USD, both exceeding 40% of the national totals. The YREB is a driving force of China’s urbanization and economic development.

As shown in Figure 1, the general norm is to classify Chongqing, Sichuan, Yunnan, and Guizhou as the upstream; Hubei, Hunan, and Jiangxi as the midstream; Anhui, Jiangsu, Zhejiang, and Shanghai as the downstream. These three regions are highlighted in different colors on the map.

### 2.2. Data Sources

2013 marks a significant turning point in the history of economic development in the YREB. It was in this year that “Relying on the golden waterway to develop the YREB” was written into the work report of the Chinese government and priority has been shifted from a more economic-oriented development strategy to a more sustainable development mode with proper emphases on ecological environment in the YREB. Given these considerations, we choose the 2013 cross-section data in the YREB to carry out our case study. The research data are collected from the “China Statistical Yearbook (2014)”, “China Water Resources Bulletin (2013)”, “China Environment Statistical Yearbook (2014)”, “Statistical Yearbook (2014)” of the 11 YREB administrative units, “Water Resources Bulletin (2013)” of the 11 YREB administrative units, “Statistical Communique on National Economic and Social Development (2013)” of the 11 YREB administrative units, and “The Yangtze River Basin and Southwest River Water Resources Bulletin (2013)”. The meteorological data such as daily maximum and minimum temperatures, and wind speed, which are used to calculate crop evapotranspiration, are taken from the National Meteorological Information Center (http://data.cma.cn/data/detail/dataCode/A.0029.0001.html), and can be accessed by registering as an authorized researcher. The crop coefficient data are taken from Chapter 6 of Crop Evapotranspiration—Guidelines for Computing Crop Water Requirements, published by the Food and Agriculture Organization (FAO) database ([25] and available at: http://www.fao.org/docrep/X0490E/x0490e0b.htm#crop%20 coefficients) but due to lack of region-specific data, these crop coefficients should be adjusted according to the growth stages of local crops [26,27,28].

As such, by investigating the crop growth of the 11 YREB administrative units in 2013, we made appropriate adjustments to the crop coefficients accordingly. Due to lack of field research data for other years, we restrict our analysis for 2013 in this research. Cultivated crops in the YREB consist of rice, middle-season rice, wheat, corn, sorghum, beans, potato, peanut, rapeseed, tobacco, vegetables, fruits and other varieties, and livestock products include beef, lamb, pork, chicken, milk, eggs, and so on. Table 1 shows the basic data of the YREB. More detailed data for water footprint accounting are given in Appendix A.

## 3. Model Construction

We first construct a generic model to calculate the current water footprints in an area and, then, employ an LAWF algorithm to optimize the water footprints. Finally, an IOWF model is furnished to analyze the impact of the LAWF scheme on the water use efficiency in each area. These models are then calibrated by using the 2013 cross-section data in the YREB. The following assumptions are made in establishing our models:

### 3.1. Assumptions

Given the actual situation of water footprint accounting, such as standards, availability of statistical data, as well as the quality of data, we make the following three assumptions:

*Assumption 1*: Theoretically, water footprints consist of blue, green, and grey components. However, given the current water resources management practice in the real world, limited data on grey water footprints are made available. In addition, scholars often differ in their opinions on a proper approach to calculating grey water footprints. As such, existing research such as Pute et al. [29], Zhuo et al. [14], and Kang et al. [30] tends to omit grey water footprints. Following the same line, we focus on blue and green water footprints and neglect grey water footprints in this study. Without causing confusion, we shall hereafter use water footprints to represent blue and green water footprints in this paper.

*Assumption 2*: We choose GDP as the key influencing factor of the LAWF model. To calibrate our model in the case study, the GDP value of administrative unit *i* is taken from the China Statistical Yearbook.

*Assumption 3*: We assume that the total water footprints consist of the primary, secondary and tertiary industries’ water footprints and other water footprints, where other water footprints account for residential water consumption and urban greening water footprints. When LAWF is entertained, this research considers only the primary, secondary and tertiary industries’ water footprints and assumes that other water footprints are not part of the LAWF process.

### 3.2. Water Footprint Accounting

As stated in Assumption 3, total water footprints are decomposed into water footprints from the primary, secondary and tertiary industries in administrative unit *i*, the primary (*WF_1i_*), secondary (*WF_2i_*), and tertiary (*WF_3i_*) industry, as well as other water footprints (*WF_4i_*) from residential water consumption and urban greening uses. Given the accounting methods differ for the primary, secondary and tertiary industries’ and other water footprints, we propose separate formulas to calculate the current water footprints to obtain the original data for further LAWF.

#### 3.2.1. Calculation of *WF*_1*i*_

CROPWAT [31] is a decision support tool developed by the Land and Water Development Division of Food and Agriculture Organization (FAO), which aims to assist in determining water demand and irrigation need for different crops based on climate conditions. The production tree method [28,32] measures virtual water content of agricultural products by establishing a tree of products. By employing the CROPWAT model and the production tree method, this paper calculates water footprints of crop cultivation and animal husbandry as follows:

(1) $$WF_{1i}=\sum_{j}\frac{10\cdot K_{ij}\cdot \sum_{d}ET_{ij}(d)\cdot S_{ij}}{G_{ij}}+\sum_{k}M_{ik}\cdot \frac{\int_{Birth}^{Slaughter}\left\{Water_{ikd}+Water_{ik,serve}+Water_{ikh}+\sum_{h}SWD(h)\times C_{ik}(h)\right\}dt}{Weight_{ik}}$$

In Equation (1), the subscripts *i*, *j*, *h* and *k* refer to administrative unit *i*, crop *j*, edible feed crop *h* and animal *k*, respectively. For crop cultivation in the first term, *K_ij_* is the average crop coefficient for growing crop *j* in administrative unit *i*. Due to lack of region-specific data in the YREB, we use the FAO recommended values combing with our investigation here. *ET_ij_* (mm/day) stands for the amount of evapotranspiration per day of growing crop *j* in administrative unit *i*. *S_ij_* (ton/year) gives the annual production of crop *j* in administrative unit *i*. *G_ij_* (ton/km^2^) signifies the yield per unit area of crop *j* in administrative unit *i*. For animal husbandry in the second term, *M_ik_* (ton/year) denotes the annual production of animal *k* in administrative unit *i*. *Water_ikd_* (m^3^/day) indicates the daily amount of water consumed by animal *k* in administrative unit *i*. *Water_ik,serve_* (m^3^/day) represents the total volume of water used to clean the animal, the range, and other services necessary to maintain the environment during the entire life span of animal *k*. In the life cycle of animal *k*, the amount of water consumption through the edible feed crop *h* consists of two parts: *Water_ikf_* (m^3^/day) stands for the actual water required for preparing the feed crop *h* and *SWD(h)* (m^3^/ton) denotes water demand of feed crop *h*. *C_ik_*(*h*) (ton/day) signifies the quantity of feed crop *h* consumed by animal *k* in administrative unit *i. Weight_ikf_* (ton) is the weight of animal *k* at the end of its life span.

#### 3.2.2. Calculation of *WF_2i_*

By following the research by Hoekstra and Chapagain [28], Chen et al. [33], and Zhao et al. [34], we take the sum of industrial water consumption and net virtual water owing to import and export of industrial products to account for the secondary industry’s water footprints. *WF_2i_* is thus calculated as:

(2) $$WF_{2i}=Q_{iMI}\cdot P_{iIO}+VW_{iI}-VW_{iO}$$

In Equation (2), *Q_iMI_* (m^3^/USD) is the water consumption for each 10,000 USD industrial output in administrative unit *i*. *P_iIO_* (USD/year) is the annual gross industrial output value (in 10,000 USD) in administrative unit *i*. *VW_iI_* (m^3^/year) is the virtual water amount due to administrative unit *i*’s imported industrial products and *VW_iO_* (m^3^/year) is the virtual water amount owing to administrative unit *i*’s exported industrial products.

#### 3.2.3. Calculation of WF_3i_

It is understandable that *WF*_3*i*_ is generally difficult to calculate. In this paper, following existing research [35,36,37], we use the tertiary industry’s water consumption for this purpose. As per the definition of “domestic water consumption” in the China Statistical Yearbook (2014), the tertiary industry’s water consumption (*WC*_3*i*_) = domestic water consumption - residential water consumption (*WC_ri_*). As such, *WF*_3*i*_ is determined as follows:

(3) $$WF_{3i}=WC_{3i}=\sum_{g}U_{ig}\cdot P_{ig}-WC_{ri}$$

In Equation (3), *WC*_3*i*_ (m^3^/year) is the annual tertiary industry’s water consumption in administrative unit *i*. *U_ig_* (m^3^/person) is the per capita water quota, *P_ig_* (person/year) is the annual population, $\sum_{g}U_{ig}\cdot P_{ig}$ is administrative unit *i*’s domestic water consumption. *WC_ri_* (m^3^/year) is the annual residential water consumption in administrative unit *i*.

#### 3.2.4. Calculation of *WF*_4*i*_

We define the sum of residential water consumption and urban greening water uses as other water footprint as shown in Equation (4):

(4) $$WF_{4i}=WC_{ri}+A_{i}\cdot \eta_{i}\cdot EP_{i}$$

In Equation (4), *WC_ri_* (m^3^/year) is the annual residential water consumption in administrative unit *i*. *A_i_* (km^2^) is the urban area of administrative unit *i*; *η_i_* is the urban green coverage excluding water surface; and *EP_i_* (m^3^/km^2^.year) is the annual evapotranspiration of plants per unit area.

### 3.3. A Lexicographic Allocation of Water Footprints (LAWF) Model

The lexicographic algorithm in this paper is adapted from the models of Luss [16,38] and Liu et al. [20,21]. Given that water footprints are shared resources, the solution process of our lexicographic minimax allocation differs from the traditional algorithm in decision variables. In the traditional algorithm, decision variables are production quantities, which consume limited resources in the production process and are suitable for enterprise production planning. However, water footprints are decision variables in our model and they are optimized by using GDP as the key influencing factor. This methodology is a proven and suitable tool for achieving equitable and efficient water resources allocation [39]. Since the lexicographic algorithm can obtain the Pareto-optimal solution with equity considerations, we propose an LAWF model to allocate water footprints with an aim to account for equity and efficiency.

In general, we assume that there are *n* entities to participate in the allocation of water footprints. In the context of YREB, *n* = 11 administrative unit. Let *WF_wi_* represent the current water footprints of industry *w* in administrative unit *i* based on Equations (1)–(3). Denote the optimized value of industry *w*’s water footprints in administrative unit *i* by $X_{wi},w\in \left\{1,2,3\right\},i\in N=\left\{1,2,\ldots ,n\right\}$. Here, *w* = 1, 2, 3 represents the primary, secondary and tertiary industry, respectively. $F^{w}(x_{wi})=\alpha_{wi}\cdot \frac{WF_{wi}-X_{wi}}{WF_{wi}},w\in \left\{1,2,3\right\},i\in N$ signifies the scarcity indicator of industry *w*’s water footprints, $F^{w}(X_{wi})<0$ indicates that administrative unit *i* has a surplus in industry *w*’s water footprints, $F^{w}(X_{wi})>0$ means that industry *w*’s water footprints in administrative unit *i* is in a shortage status, and $F^{w}(X_{wi})=0$ corresponds to a balanced water supply and demand in administrative unit *i*. To address the optimal allocation of provincial water footprints using GDP as the key influencing factor, an LAWF model is introduced as follows:

(5) $$\begin{array}{l} Lex\;\underset{x_{wi}}{\min}[F^{w}(X_{wi})]=Lex\;\underset{x_{wi}}{\min}[\alpha_{wi}\cdot \frac{WF_{wi}-X_{wi}}{WF_{wi}}]\\ s.t.\;\left\{ \begin{array}{l} \alpha_{wi}=\{\frac{GDP_{wi}}{\sum_{i}GDP_{wi}}|0<\alpha_{wi}<1\},w\in \left\{1,2,3\right\}\\ 0\leq \sum_{w\in \left\{1,2,3\right\}}\sum_{i\in N}\left(X_{wi}+WF_{4i}\right)\leq \sum_{i\in N}Q_{i}\\ {\underline{Q}}_{i}\leq \sum_{w\in \left\{1,2,3\right\}}X_{wi}+WF_{4i}\leq {\bar{Q}}_{i},\forall i\in N \end{array}\right. \end{array}$$

In Equation (5), *GDP_wi_* (USD) is industry *w*’s GDP in administrative unit *i*, *Q_i_* (m^3^) is the available water quantity in administrative unit *i*. ${\underline{Q}}_{i}$, ${\bar{Q}}_{i}$ are the lower and upper limit of the available water quantity in administrative unit *i*, respectively. The objective function is to lexicographically adjust the water footprints shortage rate in an iterative manner. Constraint 1 defines the key influencing factor (*α_wi_*) as the ratio of industry *w*’s GDP in administrative unit *i* relative to the total of industry *w*’s GDP across the 11 YREB administrative units. The next inequality constraint ensures that the sum of the lexicographically-optimized primary, secondary and tertiary industries’ water footprints and the original other water footprints (according to Assumption 3, other water footprints will not be optimized and remain as is) across the YREB is within the available water quantity in the whole area. Constraint 3 stipulates that each administrative unit’s optimized water footprints stay within the range of its available water resources. In addition, we assume that $\sum_{i\in N}{\underline{Q}}_{i}\leq \sum_{i\in N}Q_{i},i\in N$ to ensure the existence of optimal solutions and there exists at least one administrative unit *i* in water shortage from a water footprint perspective such that its original water footprints satisfy $\sum_{w\in \left\{1,2,3\right\}}WF_{wi}+WF_{4i}\geq Q_{i},i\in N$. Luss [16,38] proved that $Lex\;\min^{*}>0$ and an optimal solution exists in this case. For detailed descriptions of the solution procedure, readers are referred to Appendix B and Liu et al. [21].

### 3.4. An Input-Output Capacity of Water Footprint (IOWF) Model

The notion of input-output capacity of water footprints (IOWF) arises from the concept of water footprint intensity (WFI) [40,41]. IOWF is defined as the reciprocal of WFI. Generally, WFI characterizes water footprints consumed for each unit of GDP. It gauges water use efficiency from an input perspective and reflects a region’s water resources consumption level per unit output. Reciprocally, IOWF represents the GDP generated by each unit of water footprints. It describes water use efficiency from an output perspective and reflects a region’s water resource output level per unit input. Given that we aim to optimize water footprint allocation from an economic equity angle, we adopt IOWF as an indicator to assess the resulting LAWF scheme. IOWF is reported in the unit of USD/m^3^ and calculated as follows:

(6) $$IOWF_{i}=\frac{1}{WFI_{i}}=\frac{GDP_{i}}{WF_{i}}$$

Equation (6), *GDP_i_* is the GDP of administrative unit *i*, and *WF_i_* is its water footprints. The higher the IOWF of an administrative unit, the greater the water use efficiency. If *IOWF_wi_*, *w* = 1, 2, 3, i=1,2,…,n, is relatively high for industry *w* in administrative unit *i*, this industry is deemed a stronger industry; otherwise, it is referred to as a weaker industry from an input-output perspective.

**Figure 2:**
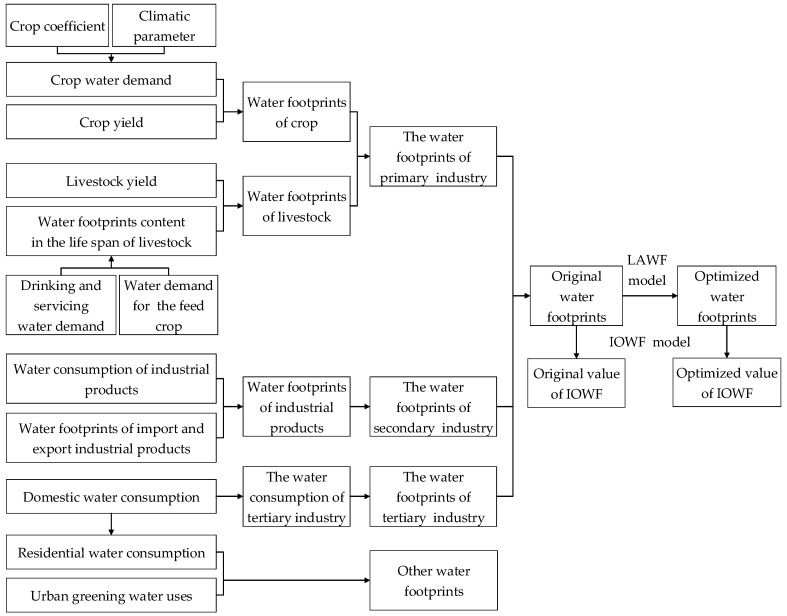
Steps in calculating water footprints and the corresponding input-output capacity.

The various steps in calculating water footprints and the corresponding input-output capacity are presented schematically in Figure 2.

## 4. Allocation Result

### 4.1. A Lexicographic Allocation Scheme of Water Footprints in the YREB

According to the “Water Resources Bulletin” of the 11 YREB administrative units, the total annual water consumption of the YREB increased from 21.85 billion m^3^ in 1998 to 265.14 billion m^3^ in 2013. Rapid economic growth and social development have significantly aggravated the load of water consumption and intensified the contradiction between water supply and demand in the YREB. Given this situation, this paper selects GDP as the key influencing factor to calibrate the LAWF model with an aim to optimize water footprint allocation from a demand management perspective.

Table 2, Table 3 and Table 4 show the iterative calculation results of the intermediate parameter values for the primary, secondary and tertiary industries’ water footprints during the lexicographic optimization process.

Allocations of the primary, secondary and tertiary industries’ water footprints follow the same lexicographic optimization process as detailed in Appendix B. Taking the primary industry’s water footprint optimization as an example, the algorithm starts with any initial estimated primary industry’s water footprint value *A*_1_ and iteratively solves the lexicographic minimax model (5) for optimal $X_{1i}$ that minimizes the absolute deviation $\left|A_{1}-\sum_{i}X_{1i}\right|$. The difference $A_{1}-\sum_{i}X_{1i}$ is then determined for this iteration. The next iteration plugs in a new trial value of *A*_1_ with an aim to decrease the absolute deviation. Table 2 illustrates the iterative process of obtaining the optimal solution and shows the values of several parameters, where $T_{1}=\sum_{i\in I}WF_{1i}-A_{1}$ stands for the difference between the current primary industry’s water footprints and the estimated value at each iteration, $R_{1}=\sum_{i\in N}(WF_{1i}/\alpha_{1i})$ gives the current primary industry’s GDP-linked water footprints, $K_{1}=T_{1}/R_{1}$ is a dimensionless ratio between the aforesaid two water footprints, and $AVG_{1}=A_{1}/\sum_{i\in N}GDP_{1i}$ gives the average primary industry’s water footprints per unit GDP across the YREB. From the iteration results in Table 2, one can see that $\left|A_{1}-\sum_{i}X_{1i}\right|$ decreases when *A*_1_ increases from 590 to 610, but the absolute deviation increases in *A*_1_ when it goes beyond 620, indicating the optimal solution is located between 610 and 620. By the iterative process, one can find that $A_{1}-\sum_{i}X_{1i}=0$ at *A*_1_ = 615.66 and the corresponding $X_{1i}$ gives us the optimal solution to this lexicographic minimax problem. Similarly, Table 3 and Table 4 present the optimization process for the secondary and tertiary industries’ water footprints. At *A*_2_ = 59.77 and *A*_3_ = 19.95, the lexicographic optimal solutions are obtained for the secondary and tertiary industries’ water footprint allocations. Subsequently, we can derive the optimized values of water footprints in the 11 YREB administrative units as shown in Table 5 and Table 6.

The original values of total water footprints in Table 5 and Table 6 are calculated by applying Equations (1)–(4) and the optimized values of the primary, secondary and tertiary industries’ water footprints are derived by plugging the corresponding original values into the LAWF model (5). The values of water footprints are expressed in billion m^3^.

**Figure 3:**
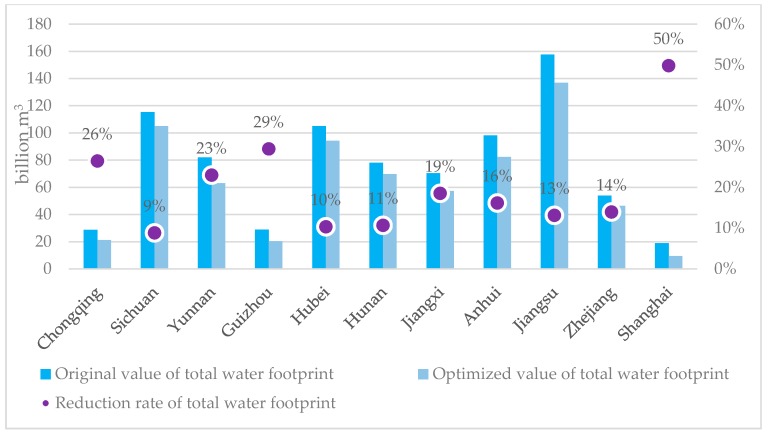
Changes of the total water footprints and the reduction rates in the YREB.

As Table 5 and Figure 3 clearly show, the optimized total water footprints of the 11 YREB administrative units are significantly reduced from the current levels with an average reduction ratio of 20.03% and a total reduction of 131.07 billion m^3^ water footprints across the whole YREB. Figure 3 also displays a clear spatial variation in the reduction rates in the 11 YREB administrative units. From a regional perspective, the average reduction ratios of total water footprints in the three segments are ranked as downstream (23.27%) > upstream (21.93%) > midstream (13.18%).

### 4.2. Input-Output Capacity of Water Footprints (IOWF) in the YREB under the Lexicographic Allocation Scheme

**Figure 4:**
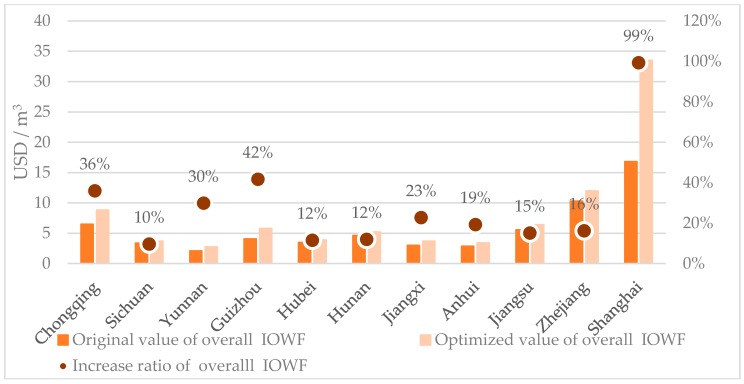
Changes of overall IOWFs and their increase ratios in the YREB.

We further explore the effectiveness and reasonableness of the LAWF allocation scheme by examining the overall IOWF metric for each YREB administrative unit. By plugging the current and optimized total water footprints into Eq. (6), one can easily verify that the overall IOWFs of the 11 YREB administrative units have been significantly improved as shown in Table 7 and Figure 4. More specifically, the original and optimized overall IOWFs for the 11 YREB administrative units are calculated as the ratio of their 2013 GDPs over the original and optimized total water footprints in the same year, respectively. Table 7 shows the results of overall IOWFs before and after the LAWF. The unit of original and optimized IOWFs is USD/m^3^.

As shown in Figure 4, the overall IOWFs of the 11 YREB administrative units show significant increases by implementing the LAWF scheme. On the other hand, the relative standing of these 11 provincial IOWFs remains the same after the LAWF scheme is applied.

Across the 11 YREB administrative units, the average increase ratio in the overall IOWFs is 28.49%. At the regional level, the increase ratios of the overall IOWFs of the three segments are ranked as downstream (37.49%) > upstream (29.30%) > midstream (15.41%). This result clearly shows that the LAWF scheme can effectively improve the overall IOWFs of the 11 YREB administrative units and the improvement tends to display spatial differences and varies from one administrative unit to the next.

### 4.3. Validation of the Model Results

Based on the optimization results of the water footprint and IOWF of each administrative unit in the YREB, this paper analyzes the validity of the model.

For the average total water footprints in 2013, the midstream (average 84.57 billion m^3^) > downstream (average 82.23 billion m^3^) > upstream (average 63.78 billion m^3^). After applying the LAWF scheme, the ranking remains intact but at lower levels as midstream (average 73.82 billion m^3^) > downstream (average 68.83 billion m^3^) > upstream (average 52.48 billion m^3^). As the correlation coefficient between the original and optimized values of the average total water footprints is 0.996 (Table 8), the 2013 data appear to be a true reflection of the water use situation in the YREB and the strong correlation indicates that the LAWF scheme effectively contains water uses without significantly changing the water use patterns in the YREB, making the scheme more receptive and implementable.

Similarly, for the original overall IOWFs in the three YREB segments, one has downstream (average 8.89 USD/m^3^) > upstream (average 4.03 USD/m^3^) > midstream (average 3.72 USD/m^3^). After applying the LAWF scheme, we confirm the same relationship at higher levels as downstream (average 13.84 USD/m^3^) > upstream (average 5.28 USD/m^3^) > midstream (average 4.26 USD/m^3^). This clearly shows that the LAWF scheme does not change the ranking of the overall IOWFs in the three YREB segments. At the same time, the original and optimized overall IOWFs in the 11 YREB administrative units are strongly and positively correlated with a correlation coefficient of 0.969 (Table 9). As such, we can conclude that the LAWF scheme can effectively strengthen the overall IOWFs without affecting their spatial patterns across the YREB.

## 5. Analysis and Discussion

The lexicographic optimization process in Section 4 indicates that the LAWF starts with accounting for current water footprints by examining water uses and comes up with reduced water demand by lowering water footprints, thereby effectively enhancing IOWFs in the YREB. Next, we will further examine different factors that affect the IOWFs of the LAWF scheme at the industrial and regional level in the YREB. Subsequently, we will explore how different regions can better exploit their industrial attributes and location characteristics to strengthen their IOWFs.

### 5.1. Analysis of Water Footprint Reductions in the YREB under the Lexicographic Allocation

**Figure 5:**
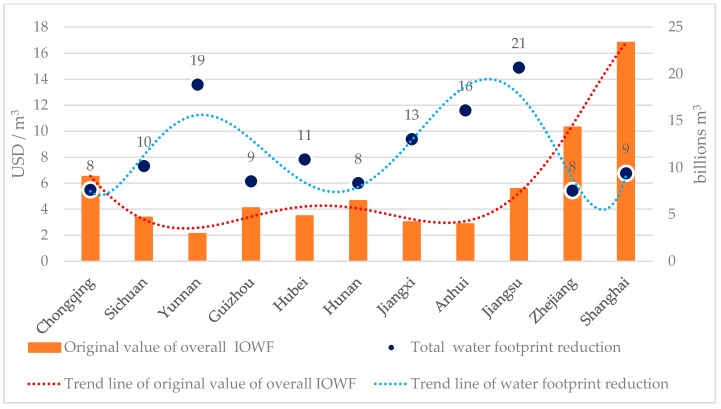
Relationship between the original overall IOWF and water footprint reduction in the YREB.

The total water footprint reductions and the original overall IOWFs are shown in Figure 5 for the 11 YREB administrative units. The original overall IOWFs are expressed in USD/m^3^. The total water footprint reduction for each administrative unit is determined as the difference between its original and optimized values of total water footprints and is expressed in billions of m^3^.

As shown in Figure 5, the two fitted trendlines show that water footprint reductions are negatively correlated with the original overall IOWFs in the YREB. The average total water footprint reduction is 11.92 billion m^3^ and the average original overall IOWF is 5.71 USD/m^3^ across the 11 YREB administrative units. From Table 7, one can see that Yunnan and Anhui are at the lower end of the original overall IOWFs (2.12 USD/m^3^ and 2.87 USD/m^3^, respectively) in these 11 administrative units, while their total water footprint reductions are relatively high at 18.86 billion m^3^ and 15.88 billion m^3^, respectively. This indicates that the LAWF scheme tends to allow an administrative unit with higher original overall IOWF to have a smaller water footprint reduction. Recall that IOWF measures water use efficiency from an output perspective, the implication is that the higher the water use efficiency, the lower the total water footprint reduction. From an economic output angle, this is a sensible strategy: For those administrative units that can make better use of scarce water resources, more water can be allocated to them with relatively smaller reduction. For those administrative units with low water use efficiency, they are required to reduce their water demand more dramatically.

The aforesaid general trend does have exceptions: Shanghai has the highest original overall IOWF (16.82 USD/m^3^), but its total water footprint reduction is not the lowest (though relatively low at 9.5 billion m^3^). Furthermore, Jiangsu has relatively high IOWF at 5.57 USD/m^3^, but its water footprint reduction is the highest at 20.68 billion m^3^. This indicates that there exist different factors that affect the water resources allocation result. As different industries tend to have different water demand patterns. This difference is influenced by industrial attributes on the one hand and by natural endowments on the other. As such, we carry out a further examination on how different industrial attributes and natural endowments affect the IOWFs of the LAWF scheme and how they can be better exploited to enhance IOWFs at the industrial and regional levels.

### 5.2. Impact Analysis of Industrial Attributes on the Iowfs of the LAWF Scheme in the YREB

**Figure 6:**
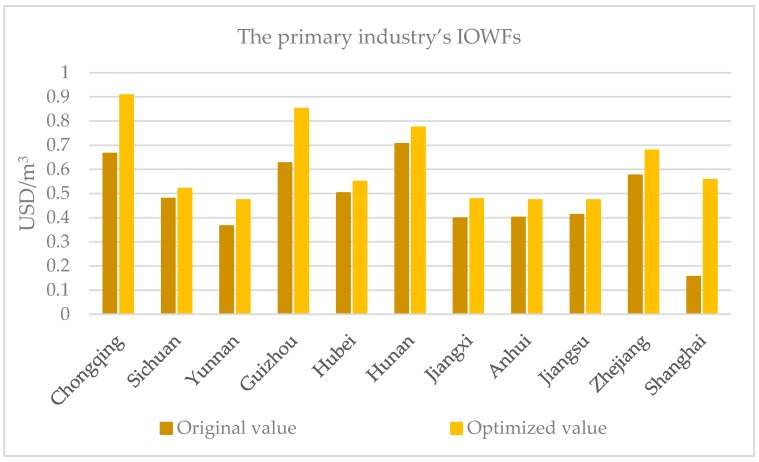

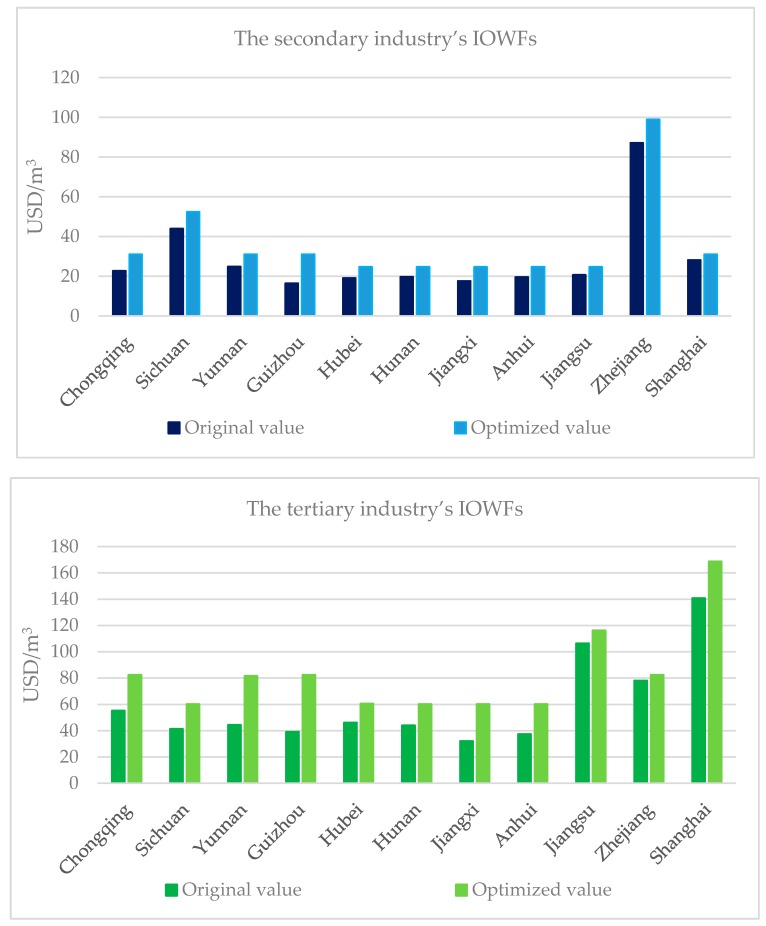
Improvement of the primary, secondary and tertiary industries’ IOWFs in the YREB.

According to the LAWF scheme, the primary, secondary and tertiary industries’ IOWFs of the 11 administrative units show significant improvement as shown in Figure 6 and Table 10. By Equation (6), the original and optimized IOWFs of the primary, secondary and tertiary industries are given by the ratios of their GDPs over the corresponding water footprints and are expressed as USD/m^3^.

As shown in Table 10, the primary, secondary and tertiary industries’ IOWFs in the three YREB segments are affected by different regional industrial attributes such as the industrial structure and spatial location. In line with Yao et al. [42] and Duarte et al.’s research on industrial site selection [43], we find that needs for factor endowments are heterogeneous in different industries in the YREB. More specifically, one observes the following:

1) *Different industries vary in their water use efficiencies as reflected by different IOWFs in the YREB.* The primary, secondary and tertiary industries’ average original IOWFs in the YREB are ranked as: tertiary industry (60.59 USD/m^3^) > secondary industry (29.15 USD/m^3^) > primary industry (0.48 USD/m^3^). It is understandable that the tertiary industry’s water use efficiency is the highest, followed by the secondary industry and lastly, by the lowest primary industry. According to the Statistical Yearbook (2014) [44], the tertiary industry mainly includes the wholesale and retail, financial, and real estate industries, the secondary industry consists of manufacturing, construction, and mining industries, and the primary industry comprises the agriculture, forestry, animal husbandry, and fishery industries. Generally speaking, from the primary industry to the secondary and tertiary industry, the water use intensity decreases while the water use efficiency increases, leading to the lowest IOWF in the primary industry, the moderate IOWF in the secondary industry, and the highest IOWF in the tertiary industry.

2) *Different industries’ IOWFs in the YREB depend on their spatial locations.* For the primary industry’s original IOWFs, midstream (0.54 USD/m^3^) > upstream (0.53 USD/m^3^) > downstream (0.39 USD/m^3^); for the secondary industry, downstream (38.94 USD/m^3^) > upstream (27.07 USD/m^3^) > midstream (18.87 USD/m^3^); for the tertiary industry, downstream (90.76 USD/m^3^) > upstream (45.19 USD/m^3^) > midstream (40.90 USD/m^3^). It is clear that the midstream has the strongest primary industry, but its secondary and tertiary industries are the weakest among the three segments of YREB; the downstream has the strongest secondary and tertiary industries, while its primary industry is the weakest; the upstream stands in the middle for primary, secondary and tertiary industries, but its primary industry’s IOWF is about at par with the midstream. This is consistent with the actual economic development situations in the YREB: The upstream and the midstream are traditional agricultural areas in China, and the midstream region is China’s critical commodity grain base. The strength of the primary industry in the midstream roots in its large-scale agricultural production. For example, its total crop cultivation area is 1.54 times that of the downstream and 1.16 times that of the upstream; its primary industry’s output is 1.32 times that of the downstream and 1.42 times that of the upstream. As for the downstream, Shanghai, Jiangsu, and Zhejiang are China’s economic powerhouses. Its strengths in the secondary and tertiary industries are related to its dense population, convenient transportation, high-tech industries, and well-developed service industries. These characteristics have enhanced the economic benefits of the secondary and tertiary industries, leading to higher water use efficiencies in these industries in the downstream. In short, the primary industry is more dependent on large-scale production, while the secondary industry needs to be close to the location of raw materials, and depends on convenient transportation and the tertiary industry relies on well-developed economy and a large service consumer group.

Comparing primary, secondary and tertiary industries’ IOWFs in the YREB before and after optimization, the LAWF scheme enhances each administrative unit’s IOWFs in each of primary, secondary and tertiary industries, but the increase rates vary across industries and administrative units. For the strongest primary industry in the midstream, its IOWF increases by 13.04%, which is the smallest among the three regions. Similarly, the downstream has the strongest secondary and tertiary industries and the corresponding increase rates stand at the lowest levels of 17.50% and 23.72% across the three regions in the YREB. At the same time, the increase rates of the weakest secondary and tertiary industries’ IOWFs are relatively high at 31.60% and 51.69% in the midstream, and the increase rate of the weakest primary industry’s IOWF comes at the highest level at 77.00% in the downstream. Therefore, the increase rates of the IOWFs for the strongest industries tend to be lower than the weakest industries as the more efficient industries tend to have smaller potentials for further improvement by implementing the LAWF scheme.

In summary, we analyze the industrial attributes of each region in the YREB and find that both industrial structure and spatial location can affect an industry’s IOWF and are the basic driving force for strengthening the IOWFs of different industries at distinct increase rates. Given that different spatial locations have distinct natural endowments, our next step is to explore how natural endowments affect the IOWFs of the LAWF scheme and put forward several policy suggestions to effectively enhance the weakest industries’ IOWFs at a regional level.

### 5.3. Analysis of Natural Endowments and Their Impact on the IOWFs of the LAWF Scheme in the YREB

In Section 5.2, we have analyzed the relationship between industrial attributes and the IOWFs from a factor endowment perspective. According to Lin and Li [45] and Li et al. [46], a region should develop more advantageous industries consistent with its factor endowment structure. They theorize that different factor endowments in different regions are conducive to distinct industrial structure. As such, industrial site selection should carefully assess the factor endowments of potential locations. 

As natural endowments are the basic element of factor endowments, we next consider natural endowments from three aspects: terrain, transportation, and climate. By synthesizing data collected from the official websites of the 11 YREB administrative units, we summarize their impact on industrial development in Table 11, where “−” stands for a negative impact and “+” means a positive impact.

As illustrated in Table 11, different YREB regions have distinct natural endowments that have significant impact on their primary, secondary and tertiary industries’ IOWFs in the YREB. Next, we will further analyze this impact from a regional economic development angle.

1) *The impact of terrain on the IOWF is mainly reflected in the industry scale*. For the upstream region, its terrain is dominated by plateaus, with a large number of karst landforms and significant rocky desertification. These topographical features make this region poor in soil and water retention, resulting in small-scale cultivation and water intensive crops. Although the agricultural output is relatively low in quantity, the general more profitable cash crops in the upstream help to enhance its primary industry’s IOWF to 0.53 USD/m^3^, comparable to the figure 0.54 USD/m^3^ in the midstream. At the same time, the terrain of the upstream has a positive impact on the secondary and tertiary industries’ IOWFs. Significant elevation variations in the upstream river system make it ideal for harnessing hydropower resources, improving the output of electricity generation, ensuring power supply in industrial development, and fostering hydropower tourism. The unique karst landscape makes the upstream a significant tourist attraction for developing its tourism industry. For the midstream region, except for the mountainous area adjacent to the upstream, its terrain is characterized by a dense river and lake network and fertile soil, which is ideal for large-scale agricultural development and makes it a key national commodity grain base. At the same time, its relatively flat terrain has laid a good foundation for developing large-scale manufacturing and service industries. For the downstream region, its terrain is dominated by plain with an extensive river and lake system. The farming land tends to be fragmented and hard for large-scale agricultural cultivation, making its primary industry’s IOWF the lowest among the three YREB regions. But the flat land is conducive to large-scale development of the secondary and tertiary industries.

2) *The primary impact of transportation on the IOWF is by affecting the logistics capacity*. In 2013, the freight volume and cargo turnover of the three YREB regions were both downstream > midstream > upstream. As the downstream is in the plain area and close to the East China Sea, its marine, land, and air transportation networks are all well connected, and the transportation infrastructure is well developed for fostering the growth of its primary, secondary and tertiary industries. For the midstream region, its flat terrain and dense road and river networks are conducive to the transportation of agricultural products and raw materials. For the upstream region, its mountainous terrain significantly restricts its logistics capacity and slows down the growth of its agriculture, manufacturing, and service industries. 

3) *The impact of climate on the IOWF is mainly to affect the cultivation type of the primary industry*. The upstream is dominated by the plateau monsoon climate. Although the vertical variation of temperature and precipitation significantly restrict the variety of agricultural crops in this region, strong solar radiation and sufficient illumination time have greatly compensated for the adverse effect of relatively low temperature due to high altitude on the crop photosynthesis and are conducive to the high yield of more profitable cash crops. On the other hand, the midstream and downstream regions are dominated by subtropical monsoon humid climate, which, on the one hand, is beneficial for developing efficient and a wide variety of agricultural crops, but on the other hand, they are prone to typhoons and other extreme weather in the summer season. Flooding disasters owing to extreme weather conditions can significantly reduce industrial output and cause extensive economic losses. In summary, terrain mainly affects the scale of the industry, transportation tends to affect the logistics capacity, and climate directly affects the type of the primary industry. Thanks to the heterogeneous influence of these natural endowment factors, the IOWF of the upstream, midstream and downstream regions show significant differences across the primary, secondary and tertiary industries.

As discussed in Section 5.2, implementing the LAWF scheme enhances the IOWFs of the weakest industries more significantly than those of the stronger industries. Since natural endowments are significant factors in influencing different industries’ IOWFs, we shall further examine how they can be exploited to strengthen the weakest industries’ IOWFs by adjusting their industrial structures. More specifically, the midstream has the lowest IOWFs in the secondary and tertiary industries across the YREB and the downstream has the lowest IOWF in the primary industry across the YREB. For the midstream, its natural endowments such as relatively flat terrain and warm climate provide great potentials for enhancing its secondary and tertiary industries’ IOWFs. By examining the three midstream administrative units’ official websites, one can see that the leading industries in Hubei include electronic information and steel metallurgy; the leading industries in Hunan consist of cultural and electronic information; and the leading industries in Jiangxi comprises mining and electronic information. To close in the IOWF gap in the secondary and tertiary industries with the upstream and downstream, it is suggested that the midstream administrative units should take advantage of the terrain and warm climate and integrate their leading industries such as electronic information and cultural industries into the tourism and services industry. In addition, it is also recommended that this region improve its transportation infrastructure to accelerate the growth of its secondary and tertiary industries, thereby enhancing their IOWFs. For the downstream, its friendly terrain and convenient transportation furnish potentials for enhancing its primary industry’s IOWF. We recommend that the advantages of terrain and transportation should be fully exploited to increase its agricultural output and preventive measures against extreme weather should be actively taken to reduce agricultural losses. More specifically, the first suggestion is that the flood irrigation mode should be converted to sprinkler or drip irrigation to reduce agricultural water footprints. The second suggestion is to promote integration of the primary, secondary and tertiary industries to develop modern agriculture and increase the added value of agriculture. The last point is to improve agricultural disaster prevention and mitigation, thereby reducing the primary industry’s economic losses due to extreme weather such as typhoon and flooding and enhancing its IOWF.

In summary, we analyze the natural endowments in the YREB from the terrain, transportation, and climate perspectives and examine their heterogeneous impact on the primary, secondary and tertiary industries’ IOWFs. Capitalizing on this analysis, we put forward several policy suggestions for different regions to enhance the IOWFs of the weakest industries across the three YREB regions by exploiting their natural endowments, thereby facilitating sustainable economic growth in the YREB.

## 6. Conclusions

Based on the lexicographical optimization theory, this paper adopts GDP as the influencing factor to construct an LAWF and an IOW model to optimize water resources allocation with equity and efficiency considerations. By applying the proposed approach to a case study of allocating water footprints in the YREB, we derive the following results: Firstly, given available data and in line with existing research, we obtain a proper accounting of the primary, secondary and tertiary industries’ water footprints in the YREB based on the 2013 cross-sectional data. Secondly, an optimal water footprint allocation scheme is derived by using the LAWF model, which can significantly reduce water footprints in each of the 11 YREB administrative units and strengthen their IOWFs. More specifically, the average reduction ratio of total water footprints in the YREB is 20.03%, and the average increase ratio of the overall IOWFs is 28.49%. Thirdly, industrial attributes and natural endowments are the basic driving forces for strengthening the IOWFs of different industries at distinct increase rates. Natural endowment factors such as terrain, transportation, and climate can be properly exploited to strengthen the weakest industries’ IOWFs. It is concluded that this optimal water footprint allocation scheme can effectively alleviate the contradiction between water supply and demand and help to improve water use efficiency for sustainable economic growth in the YREB.

This research has its limitations and different extensions can be considered. First, due to lack of reliable data, we only adjust the crop coefficient data according to the growth stages of local crops in 2013, allowing us to conduct our analysis based on the 2013 cross-sectional data. To examine the robustness of our result, it is necessary to conduct more field research to collect region-specific data in other years so that we can conduct a more thorough analysis of the optimal LAWF allocation scheme. Alternatively, we may select a water footprint accounting method that can work with limited data, such as water footprint accounting based on input-output tables. Second, to focus on the impact of GDP on water resources allocation, we neglect the influence of other factors such as population and land area. A more sensible future study is to take a comprehensive approach for equitable allocation of water resources under the influence of multiple factors. Third, grey water footprints should be incorporated into future research by collecting reliable data for the YREB.

## Figures and Tables

**Table 1:** The basic data of the YREB in 2013.

Administrative Units	Total GDP (10^8^ USD)	Primary Industry GDP (10^8^ USD)	Secondary Industry GDP (10^8^ USD)	Tertiary Industry GDP (10^8^ USD)	Available Water Resources (billion m^3^)	Residential Water Consumption (billion m^3^)
Chongqing	1876.99	150.78	948.81	777.39	47.43	0.51
Sichuan	3894.47	508.02	2013.77	1372.68	247.03	0.95
Yunnan	1738.21	281.08	730.80	726.34	170.67	0.32
Guizhou	1187.41	152.61	481.04	553.76	75.94	0.25
Hubei	3658.34	459.46	1805.04	1393.84	79.01	1.06
Hunan	3633.60	459.62	1708.02	1465.96	158.20	0.86
Jiangxi	2126.40	242.69	1137.67	746.04	142.40	0.43
Anhui	2823.47	348.22	1542.91	932.34	58.56	0.61
Jiangsu	8773.69	540.71	4314.64	3918.33	28.35	1.60
Zhejiang	5571.41	264.66	2735.64	2571.11	93.13	1.09
Shanghai	3203.59	19.17	1190.52	1993.90	2.80	1.02

**Table 2:** Intermediate parameter values of the primary industry’s water footprints during the LAWF.

Iteration Process	*A*_1_ (billion m^3^)	$A_{1}-\sum_{i}X_{1i}$ (billion m^3^)	*T*_1_ (billion m^3^)	*R*_1_ (billion m^3^)	*K*_1_	*AVG*_1_ (m^3^/USD)
1	590	−21.50	134.08	9226.29	0.0145	1.7216
2	600	−8.10	124.08	9226.29	0.0134	1.7508
3	610	−2.92	114.08	9226.29	0.0124	1.7800
4	620	2.28	104.08	9226.29	0.0113	1.8092
Optimized value	615.66	0	108.42	9226.29	0.0118	1.7965

**Table 3:** Intermediate parameter values of the secondary industry’s water footprints during the LAWF.

Iteration Process	*A*_2_ (billion m^3^)	$A_{2}-\sum_{i}X_{2i}$ (billion m^3^)	*T*_2_ (billion m^3^)	*R*_2_ (billion m^3^)	*K*_2_	*AVG*_2_ (m^3^/USD)
1	57	−1.27	18.11	879.94	0.0206	0.0306
2	58	−0.85	17.11	879.94	0.0195	0.0312
3	59	−0.40	16.11	879.94	0.0184	0.0317
4	61	0.91	14.11	879.94	0.0161	0.0328
Optimized value	59.77	0	15.34	879.74	0.0174	0.0321

**Table 4:** Intermediate parameter values of the tertiary industry’s water footprints during the LAWF.

Iteration Process	*A*_3_ (billion m^3^)	$A_{3}-\sum_{i}X_{3i}$ (billion m^3^)	*T*_3_ (billion m^3^)	*R*_3_ (billion m^3^)	*K*_3_	*AVG*_3_ (m^3^/USD)
1	17	−0.74	10.26	363.81	0.02819	0.0103
2	18	−0.60	9.26	363.81	0.02544	0.0109
3	19	−0.37	8.26	363.81	0.02269	0.0115
4	20	0.02	7.26	363.81	0.01994	0.0122
Optimized value	19.95	0	7.30	363.81	0.02007	0.0121

**Table 5:** Total water footprints in the YREB (unit: billion m^3^).

Region	Administrative Units	Original Total Water Footprints	Optimized Total Water Footprints	Total Water Footprints Reduction	Reduction Rate of Total Water Footprints		
					Provincial	Regional	YREB’s
Upstream	Chongqing	28.83	21.20	7.63	26.46%	21.93%	20.03%
	Sichuan	115.29	105.12	10.17	8.82%		
	Yunnan	82.00	63.14	18.86	23.00%		
	Guizhou	29.00	20.47	8.53	29.42%		
Midstream	Hubei	105.10	94.23	10.87	10.34%	13.18%	
	Hunan	78.21	69.85	8.36	10.69%		
	Jiangxi	70.41	57.37	13.04	18.53%		
Downstream	Anhui	98.25	82.37	15.88	16.16%	23.27%	
	Jiangsu	157.59	136.90	20.69	13.13%		
	Zhejiang	54.02	46.48	7.54	13.96%		
	Shanghai	19.04	9.55	9.49	49.83%		

**Table 6:** The primary, secondary and tertiary industries’ water footprints in the YREB (unit: billion m^3^).

Administrative Units	Original Value of the Primary Industry’s Water Footprints	Original Value of the Secondary Industry’s Water Footprints	Original Value of the Tertiary Industry’s Water Footprints	Optimized Value of the Primary Industry’s Water Footprints	Optimized Value of the Secondary Industry’s Water Footprints	Optimized Value of the Tertiary Industry’s Water Footprints
Chongqing	22.67	4.16	1.40	16.61	3.05	0.94
Sichuan	106.05	4.57	3.30	97.64	3.84	2.27
Yunnan	76.92	2.93	1.63	59.39	2.35	0.89
Guizhou	24.36	2.91	1.41	17.93	1.55	0.67
Hubei	91.55	9.41	3.01	83.53	7.29	2.30
Hunan	65.11	8.66	3.32	59.40	6.89	2.43
Jiangxi	61.03	6.43	2.31	50.91	4.59	1.24
Anhui	86.87	7.88	2.48	73.57	6.22	1.55
Jiangsu	131.24	20.80	3.68	114.25	17.42	3.37
Zhejiang	45.98	3.14	3.29	38.99	2.76	3.12
Shanghai	12.30	4.22	1.42	3.44	3.82	1.18

**Table 7:** Total IOWFs in the YREB.

Region	Administrative Units	Original Value of overall IOWF (USD/m^3^)	Optimized Value of overall IOWF (USD/m^3^)	Increase Ratio of overall IOWF		
				Provincial	Regional	YREB’s
Upstream	Chongqing	6.51	8.85	35.98%	29.30%	28.49%
	Sichuan	3.38	3.70	9.68%		
	Yunnan	2.12	2.75	29.87%		
	Guizhou	4.09	5.80	41.68%		
Midstream	Hubei	3.48	3.88	11.53%	15.41%	
	Hunan	4.65	5.20	11.97%		
	Jiangxi	3.02	3.71	22.74%		
Downstream	Anhui	2.87	3.43	19.28%	37.49%	
	Jiangsu	5.57	6.41	15.12%		
	Zhejiang	10.31	11.99	16.22%		
	Shanghai	16.82	33.53	99.34%		

**Table 8:** Correlation coefficient between the original and optimized values of the total water footprint.

Variable		Original Value of the Total Water Footprint	Optimized Value of the Total Water Footprint
Original value of the total water footprint	Pearson Correlation	1	0.996 **
	Sig. (2-tailed)		0.000
	N	11	11
Optimized value of the total water footprint	Pearson Correlation	0.996 **	1
	Sig. (2-tailed)	0.000	
	N	11	11

** Correlation is significant at the 0.01 level (2-tailed).

**Table 9:** Correlation coefficient between the original and optimized values of the overall IOWFs.

Variable		Original Value of the Overall Lowfs	Optimized Value of the Overall Lowfs
**Original value of the overall IOWFs**	Pearson Correlation	1	0.969 **
	Sig. (2-tailed)		0.000
	N	11	11
Optimized value of the overall IOWFs	Pearson Correlation	0.969 **	1
	Sig. (2-tailed)	0.000	
	N	11	11

** Correlation is significant at the 0.01 level (2-tailed).

**Table 10:** The primary, secondary and tertiary industries’ IOWFs in the YREB (unit: USD/m^3^).

Region	Original Value of the Primary Industry’s IOWF	Original Value of the Secondary Industry’s IOWF	Original Value of the Tertiary Industry’s IOWF	Optimized Value of the Primary Industry’s IOWF	Optimized Value of the Secondary Industry’s IOWF	Optimized Value of the Tertiary Industry’s IOWF
Upstream	0.53	27.07	45.19	0.69	36.47	76.72
Midstream	0.54	18.87	40.90	0.60	24.77	60.48
Downstream	0.39	38.94	90.76	0.55	44.92	106.95
Average	0.48	29.15	60.59	0.61	36.35	83.28

**Table 11:** Natural endowments and their impact on industrial development in the YREB.

Region	Industry	Terrain	Transportation	Climate
Upstream	Primary industry	**−**	**−**	**+**
	Secondary industry	**+**	**−**	**+**
	Tertiary industry	**+**	**−**	**+**
Midstream	Primary industry	**+**	**+**	**−**
	Secondary industry	**+**	**−**	**+**
	Tertiary industry	**+**	**−**	**+**
Downstream	Primary industry	**+**	**+**	**−**
	Secondary industry	**+**	**+**	**+**
	Tertiary industry	**+**	**+**	**+**

**Figure A1:**
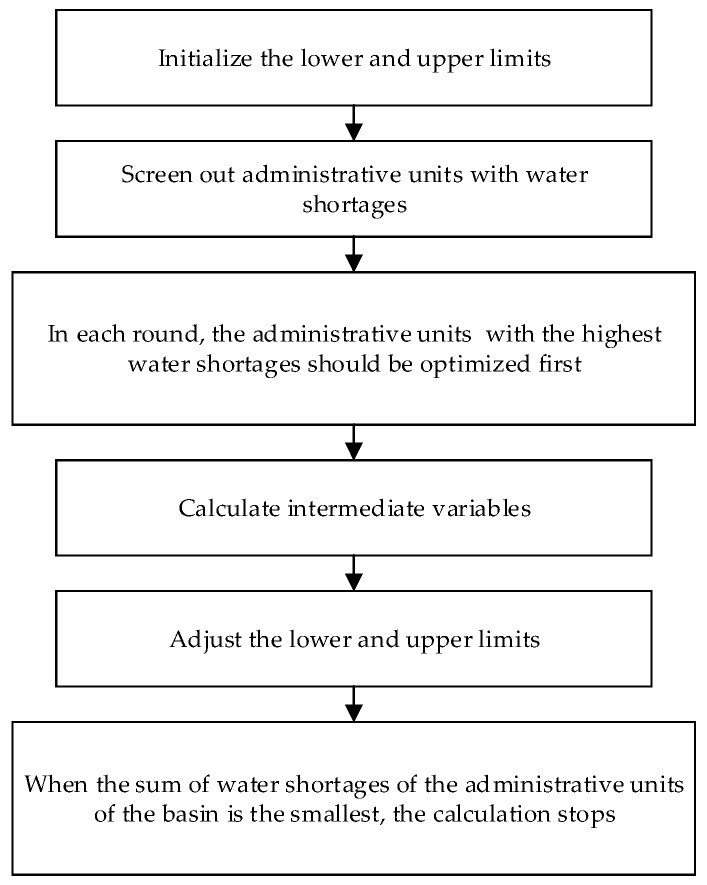
Calculation procedure.

## Appendix A. Data for Water Footprint Accounting

The tables below show the data of the primary, secondary and tertiary industries, domestic and urban greening water footprints of the 11 YREB administrative units. Table A1 shows the calculation of the primary industry’s water footprints, including cultivated crops and livestock husbandry water footprints. Table A2 lists the calculation of the secondary industry’s water footprints based on industrial production and inter-provincial trade. Table A3 displays the calculation of the tertiary industry’s water footprints. Table A4 gives the calculation of residential water consumption and urban greening water footprints of the 11 YREB administrative units.

**Table A1:** The data of the primary industry’s water footprints in the YREB.

Item	Chongqing	Sichuan	Yunnan	Guizhou	Hubei	Hunan	Jiangxi	Anhui	Jiangsu	Zhejiang	Shanghai
Wheat	3.92	48.45	7.59	5.77	52.10	1.39	0.34	187.81	214.76	3.53	0.00
Barley	0.00	0.00	0.00	0.00	0.00	0.00	0.00	0.00	2.42	0.50	0.00
Broad bean	0.00	0.00	0.00	0.00	0.00	0.00	0.00	0.00	1.88	1.24	0.00
Paddy	58.36	181.29	53.46	36.85	231.38	330.44	284.57	70.84	80.73	96.89	0.00
Maize	16.78	45.74	53.53	19.97	19.49	13.14	0.91	34.93	17.75	2.41	0.00
Sorghum	0.14	0.00	0.00	0.00	0.00	0.00	0.00	0.00	0.00	0.00	0.00
Potato	7.70	9.11	7.53	7.64	3.44	5.32	0.00	1.42	1.40	1.78	0.00
Soybean	7.55	10.13	3.87	1.34	3.76	0.00	6.45	24.62	13.17	4.94	29.45
Cotton	0.00	1.37	0.00	0.00	54.34	23.13	15.47	30.23	24.32	3.39	0.49
Peanut	2.38	13.34	2.08	2.00	10.83	5.52	11.25	23.49	9.42	1.63	0.00
Rapeseed	7.38	43.69	12.06	13.74	26.05	29.58	14.36	27.57	26.62	7.44	0.36
Sesame	0.00	6.11	0.00	0.00	15.65	1.78	4.40	8.07	0.04	0.02	0.00
Sugarcane	0.00	5.98	204.32	17.04	3.48	12.01	8.15	0.00	0.83	0.00	0.00
Mint	0.00	0.00	0.00	0.00	0.00	0.00	0.00	0.00	0.00	0.00	0.00
Vegetables	21.24	225.26	42.26	27.01	49.74	49.37	25.65	0.00	256.65	32.04	7.17
Tobacco leaf	0.00	0.95	9.76	0.79	0.79	1.24	0.25	0.21	0.00	0.02	0.00
Melon and fruit	9.66	26.59	38.71	8.72	6.67	9.22	11.39	15.10	89.04	17.20	2.00
Tea leaf	0.00	0.92	46.04	0.54	0.00	0.00	0.00	0.64	0.00	0.00	0.00
Cultivated crops’ WF (100 million m^3^)	135.09	618.93	481.21	141.41	477.71	482.13	383.19	424.94	739.01	173.04	39.47
**Livestock products**											
Pork	40.16	181.14	166.58	59.76	168.11	156.46	88.29	114.80	125.27	78.09	11.12
Beef	10.74	58.32	68.17	21.00	40.66	11.82	26.42	36.56	8.37	10.58	0.75
Lamb	0.00	23.44	13.18	2.96	15.36	0.66	1.50	29.41	17.27	11.48	0.72
Poultry	16.50	82.29	0.00	10.60	60.29	0.00	55.17	96.37	187.47	121.44	13.54
Honey	0.53	0.95	0.11	0.05	0.55	0.00	0.36	0.49	0.13	2.41	0.00
Egg	21.78	72.30	21.81	6.50	146.36	0.00	50.95	124.91	212.87	53.41	6.83
Milk	1.46	19.82	18.15	1.31	6.47	0.00	4.45	41.18	22.04	7.43	50.57
Cocoon	0.38	3.32	0.00	0.00	0.00	0.00	0.00	0.00	0.00	1.94	0.00
Livestock products’ WF (100 million m^3^)	91.55	441.57	288.00	102.17	437.81	168.94	227.14	443.72	573.43	286.78	**83.53**
WF_1i_ (100 million m^3^)	226.65	1060.50	769.21	243.58	915.52	651.07	610.33	868.66	1312.44	459.83	**123.00**

**Table A2:** The data of the secondary industry’s water footprints in the YREB.

Source	Chongqing	Sichuan	Yunnan	Guizhou	Hubei	Hunan	Jiangxi	Anhui	Jiangsu	Zhejiang	Shanghai
Industrial output value (100 million USD)	948.81	2013.77	730.80	481.04	1805.04	1708.02	1137.67	1542.91	4314.64	2735.64	1190.52
Industrial water consumption (100 million m^3^)	36.7	44.7	24.6	27.7	90.2	87.7	61.3	91.2	238	55.7	67.2
Product WF (100 million m^3^)	36.7	44.7	24.6	27.7	90.2	87.7	61.3	92.7	238	55.7	66.2
Import industrial virtual water (100 million m^3^)	42.06	30.5	24.16	26.18	46.12	37.19	49.15	39.18	46.18	40.19	34.19
Export industrial virtual water (100 million m^3^)	37.16	29.46	19.46	24.75	42.18	38.32	46.15	51.63	76.19	64.53	59.15
Industrial trade water footprint (100 million m^3^)	4.9	1.04	4.7	1.43	3.94	−1.13	3	−12.45	−30.01	−24.34	−24.96
WF_2i_ (100 million m^3^)	41.6	45.74	29.3	29.13	94.14	86.57	64.3	78.75	207.99	31.36	42.24

**Table A3:** The data of the tertiary industry’s water footprints in the YREB.

Category	Chongqing	Sichuan	Yunnan	Guizhou	Hubei	Hunan	Jiangxi	Anhui	Jiangsu	Zhejiang	Shanghai
Domestic water consumption	19.10	42.50	19.50	16.60	40.70	41.80	27.40	30.90	52.80	43.80	24.40
Residential water consumption	5.06	9.45	3.18	2.52	10.62	8.58	4.26	6.13	15.99	10.90	10.24
WF_3i_ (100 million m^3^)	14.04	33.05	16.32	14.08	30.08	33.22	23.14	24.77	36.81	32.90	14.16

**Table A4:** The data of other water footprints in the YREB.

Category	Chongqing	Sichuan	Yunnan	Guizhou	Hubei	Hunan	Jiangxi	Anhui	Jiangsu	Zhejiang	Shanghai
Residential water consumption	5.06	9.45	3.18	2.52	10.62	8.58	4.26	6.13	15.99	10.90	10.24
Urban greening water recharge	0.90	4.20	2.0	0.70	0.60	2.70	2.10	4.20	2.70	5.20	0.80
WF_4i_ (100 million m^3^)	5.96	13.65	5.18	3.22	11.22	11.28	6.36	10.33	18.69	16.10	11.04

## Appendix B. A Lexicographic Algorithm

To solve model (5), the lexicographic algorithm in Luss [16,38] and Liu et al. [20,21] is employed. To make the paper self-contained, the algorithm therein is replicated and adapted below. The algorithm follows the basic idea in Luss [16,38]. Their original concern is to address resource allocation in producing multiple products. In this paper, fair allocation of provincial water footprints under limited water supply constraints can be characterized as a single resource allocation in a single period with multiple stakeholders. The key to tackling a lexicographic minimax problem is to solve the following minimax problem successively by using the lexicographic method. In Equation (A1), *L_wi_* and *U_wi_* are the lower and upper limits of the optimized value of industry *w*’s water footprints in administrative unit *i*, respectively.

(A1) $$\begin{array}{l} P*=Min[\underset{i\in I}{Max}(\alpha_{wi}\cdot \frac{WF_{wi}-X_{wi}}{WF_{wi}})],w\in \left\{1,2,3\right\}\\ s.t.\;\sum_{i\in I}\alpha_{wi}\cdot \frac{WF_{wi}-X_{wi}}{WF_{wi}}\leq Q_{i},\;\forall i\in I\\ \;L_{wi}\leq X_{wi}\leq U_{wi},\forall i\in I \end{array}$$

The procedure can be described as follows:
